# Surgical management of a patient with anomalous origin of the left circumflex coronary artery undergoing aortic and mitral valve surgery

**DOI:** 10.1186/s44215-025-00215-4

**Published:** 2025-07-15

**Authors:** Risako Kojima, Koji Furukawa, Shohei Hiromatsu, Kousuke Mori, Ayaka Iwasaki, Sakaguchi Shuhei, Hirohito Ishii

**Affiliations:** Division of Cardiovascular Surgery, Department of Surgery, Faculty of Medicine, University of Miyazaki, 5200 Kiyotakecho Kihara, Miyazaki-City, Miyazaki 889-1692 Japan

**Keywords:** Anomalous origin of the coronary artery, Anomalous origin of the left circumflex coronary artery, Bicuspid aortic valve, Valve surgery, Coronary artery bypass grafting

## Abstract

**Background:**

The anomalous origin of the left circumflex coronary artery is rare and, when isolated, typically has minimal pathological significance. However, it can cause damage or compression of the coronary artery during aortic and mitral valve surgery.

**Case presentation:**

The patient was a 34-year-old male diagnosed with severe aortic regurgitation due to a bicuspid aortic valve following infective endocarditis at the mitral valve. He was referred to our hospital owing to worsening heart failure. Preoperative evaluation revealed a mitral valve aneurysm and an anomalous left circumflex coronary artery originating from the right coronary artery and running posteriorly along the aortic valve annulus. During surgery, dissection of the anomalous left circumflex coronary artery was challenging. Mitral valve aneurysm repair and aortic valve replacement were performed. For the aortic valve replacement, a 23-mm St. Jude Medical Regent valve, one size smaller than optimal, was secured in the supra-annular position. Additionally, a coronary artery bypass graft was performed on the distal circumflex artery using a saphenous vein graft. The patient experienced no ischemic myocardial damage and was discharged in stable condition on postoperative day 14.

**Conclusions:**

The anomalous origin of the left circumflex coronary artery should be recognized, and appropriate measures must be taken during valve surgery. Preemptive coronary artery bypass grafting is a reasonable option for patients undergoing aortic and mitral valve surgeries.

**Supplementary Information:**

The online version contains supplementary material available at 10.1186/s44215-025-00215-4.

## Background

An anomalous origin of the coronary artery occurs in 1–2% of patients undergoing coronary examinations [1–4]. Among these, the anomalous origin of the left circumflex coronary artery (AOLCX) from the right sinus of Valsalva or the right coronary artery (RCA) is the most prevalent. This condition is typically considered benign, as it rarely leads to sudden death [2, 4–7]. However, coronary blood flow may be compromised by suturing or compression of a prosthetic valve, potentially resulting in myocardial infarction [1–7]. We report the case of a middle-aged male with AOLCX who underwent aortic valve (AV) and mitral valve (MV) surgery, accompanied by preemptive coronary artery bypass grafting (CABG).

## Case presentation

The patient was a 34-year-old male who had been diagnosed with aortic regurgitation (AR) at 15 years of age but did not receive follow-up care. At 32 years of age, he experienced a splenic infarction and was diagnosed with MV infective endocarditis. Although the infection was managed with antibiotics, severe AR recurred. He subsequently developed symptoms of heart failure and was referred to our hospital for evaluation.

**Fig. 1:**
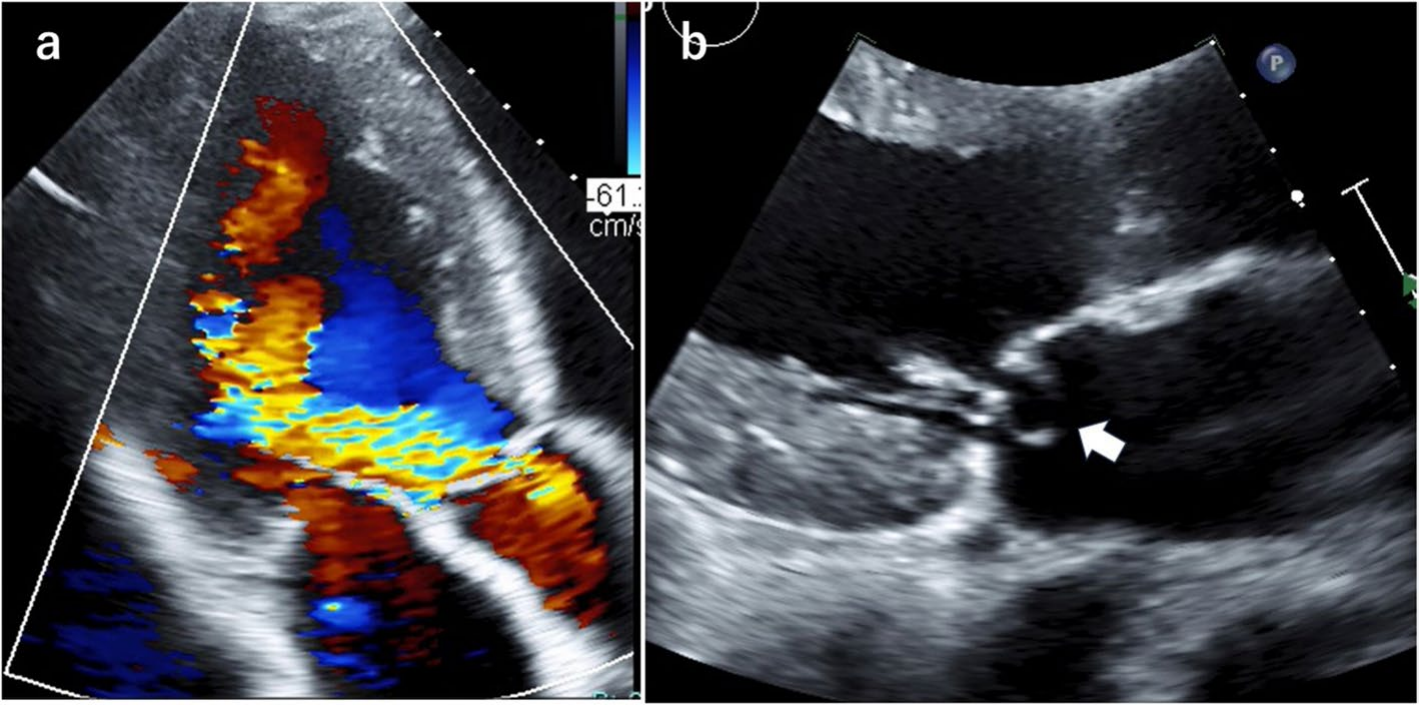
Transthoracic echocardiograms showing severe eccentric aortic regurgitation (**a**) and an anterior mitral valve leaflet aneurysm (white arrow) (**b**)

**Fig. 2:**
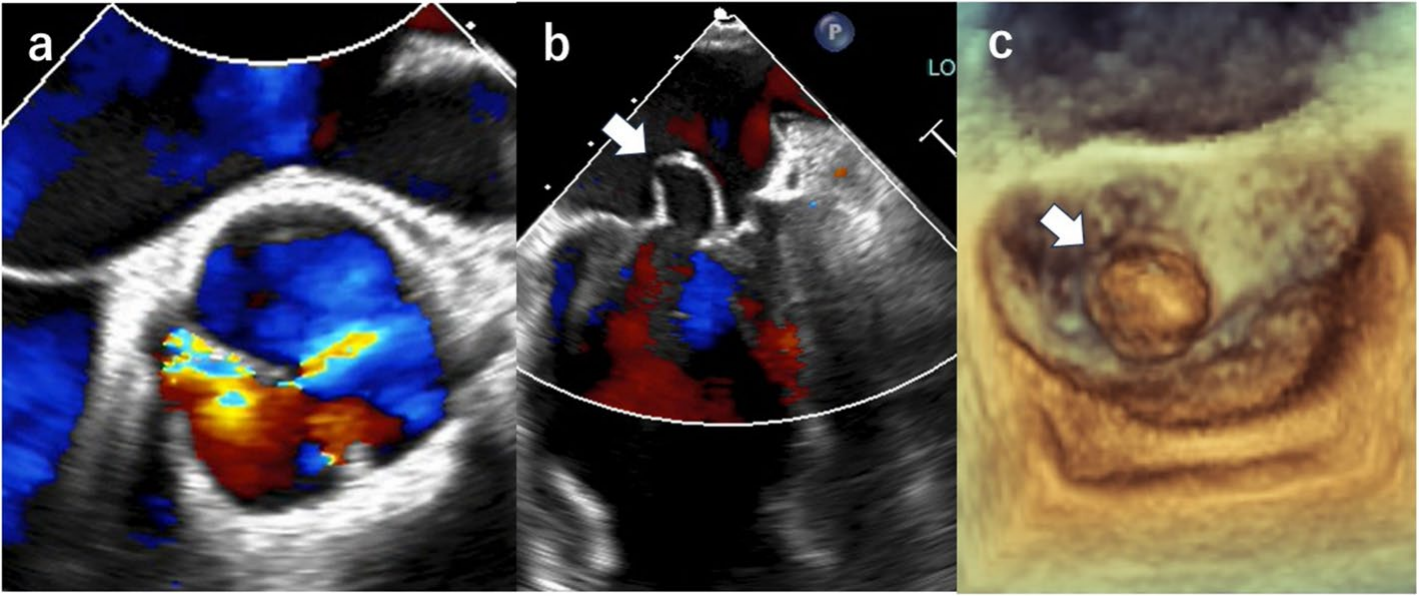
Transesophageal echocardiograms showing a Sievers Type I bicuspid aortic valve with fused left and right coronary cusps and aortic regurgitation (**a**) and an approximately 1.2-cm aneurysm of the anterior mitral valve leaflet (white arrow) (**b** and **c**)

Upon admission, the patient’s height (170 cm), weight (60 kg), blood pressure (125/60 mmHg), and heart rate (64 beats/min) were recorded. Auscultation revealed a to-and-fro murmur in the second intercostal space at the right sternal border. Chest radiography revealed a cardiothoracic ratio of 58%. Electrocardiography showed sinus rhythm and elevated left ventricular voltage (SV1 + RV5 = 4 mV). Transthoracic echocardiography revealed severe eccentric AR and an anterior leaflet MV aneurysm without mitral regurgitation (MR) (Fig. 1a and b). The left ventricular systolic and diastolic diameters were 76 mm and 45 mm, respectively, with a left ventricular ejection fraction of 66%. Transesophageal echocardiography revealed Sievers Type I bicuspid AV (BAV) with fused left and right coronary cusps and severe AR (Fig. 2a). An approximately 1.2-cm MV aneurysm was noted in the A2 clear zone of the anterior MV (Fig. 2b and c), with no evidence of aneurysm perforation into the left atrium, chordae tendineae rupture, or leaflet prolapse (Fig. 2b).

**Fig. 3:**
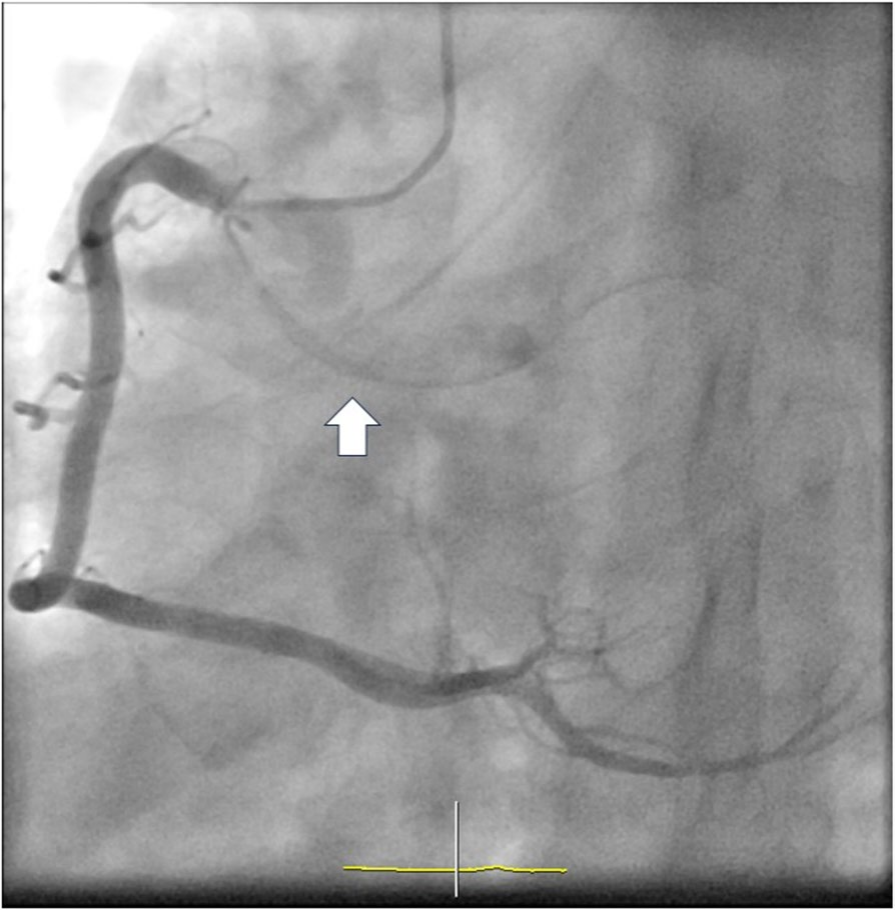
Coronary angiogram showing the left circumflex coronary artery arising near the origin of the right coronary artery (white arrow)

**Fig. 4:**
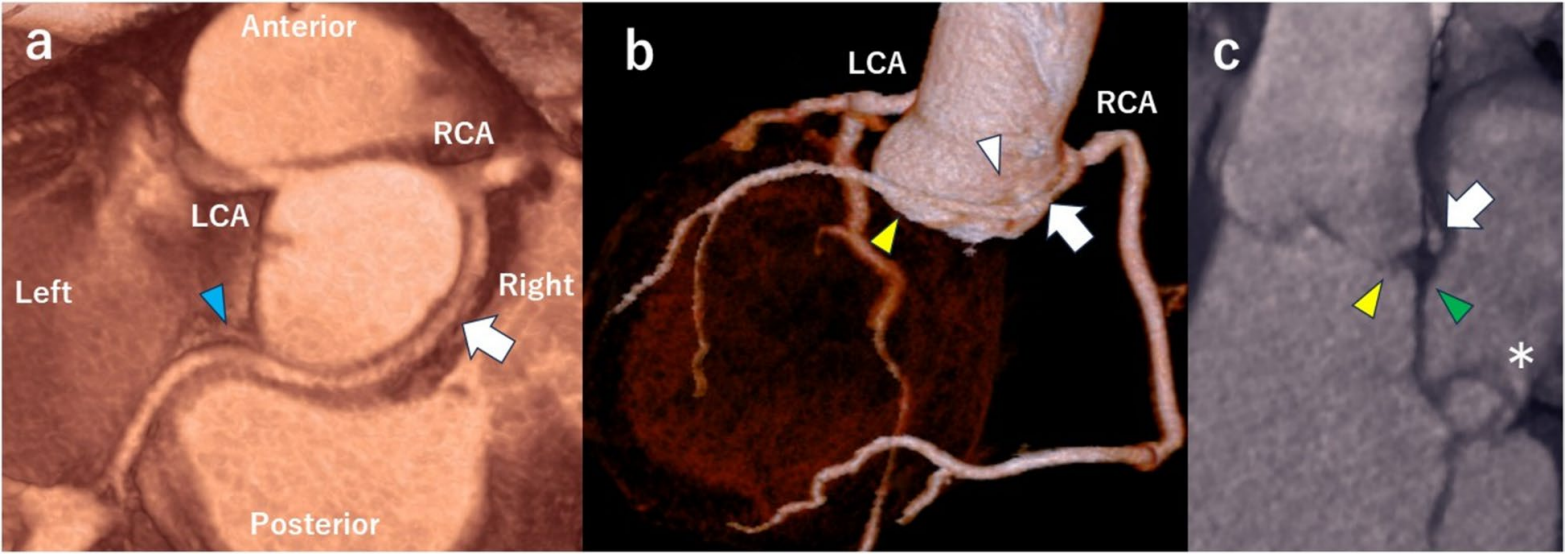
Preoperative computed tomography showing that the left circumflex coronary artery (white arrow) originated from the right coronary artery (**a** and **b**). It traversed the commissures of the right and noncoronary cusps of the aortic valve (white arrowhead) (**b**) and extended between the aorta and the right and left atria (**a** and **b**). The artery then continued to the right fibrous trigone (blue arrowhead), reaching the posterior atrioventricular groove (**a**). Between the aorta and the left atrium, it passed slightly above the aortic valve annulus (yellow arrowhead) and the mitral valve annulus (green arrowhead) (**b** and **c**). Additionally, an anterior mitral valve aneurysm (asterisk) was observed (**c**). *LCA*, left coronary artery; *RCA*, right coronary artery

Coronary angiography revealed no significant stenosis; however, the left circumflex coronary artery (LCX) bifurcated near the origin of the RCA (Fig. 3). Multidetector computed tomography (CT) demonstrated that the LCX originated from the RCA, passed between the aorta and the right and left atria, continued to the right fibrous trigone, and reached the posterior atrioventricular groove to perfuse the left ventricular lateral wall (Fig. 4a and b). Specifically, the LCX bifurcated from the proximal RCA along the aortic wall (Fig. 4a) and crossed the right and noncoronary cusp commissures of the AV (Fig. 4b). It passed slightly above the AV and MV annulus between the aorta and left atrium (Fig. 4b and c). We did not perform additional tests to assess myocardial ischemia, as no findings were suggestive of significant stenosis or an intramural course in the AOLCX.

Median sternotomy was performed under general anesthesia, and cardiopulmonary bypass was initiated via cannulation of the ascending aorta, superior vena cava, and inferior vena cava. An aortic cross-clamp was applied, and cardiac arrest was induced using selective antegrade blood cardioplegia. The roofs of the right and left atria were dissected from the aortic root, revealing an anomalous LCX covered by a layer of aortic adventitia; however, it was too close to the wall of the sinus of Valsalva for dissection. The AV was bicuspid, with fused left and right coronary cusps and no signs suggesting infection. The raphe at the fused cusps was dissected from the sinus of Valsalva, but the cusps were only 12 mm high, complicating AV repair.

Access to the MV was obtained through a right-sided left atrial incision. An MV aneurysm was identified in the clear zone of the anterior leaflet without evidence of active infection (Additional File 1a). The aneurysm was resected, leaving a suture margin, and direct suture repair was performed using 4–0 polypropylene sutures without mitral annuloplasty (Additional File 1b). After the left atriotomy was repaired, CABG was performed to the obtuse marginal branch of the LCX, with the saphenous vein harvested using the conventional open technique and dilated manually with saline.

The AVs were excised, and a 23-mm St. Jude Medical Regent valve (St. Jude Medical Inc., St. Paul, MN, USA), one size smaller than the appropriate size, was implanted in the supra-annular position. A central anastomosis of the CABG to the ascending aorta was then performed. The aortic clamp was released, and transesophageal echocardiography confirmed no issues with the repair site or cardiac wall motion. Weaning from cardiopulmonary bypass was uneventful, with graft flow measured at 30 mL/min.

**Fig. 5:**
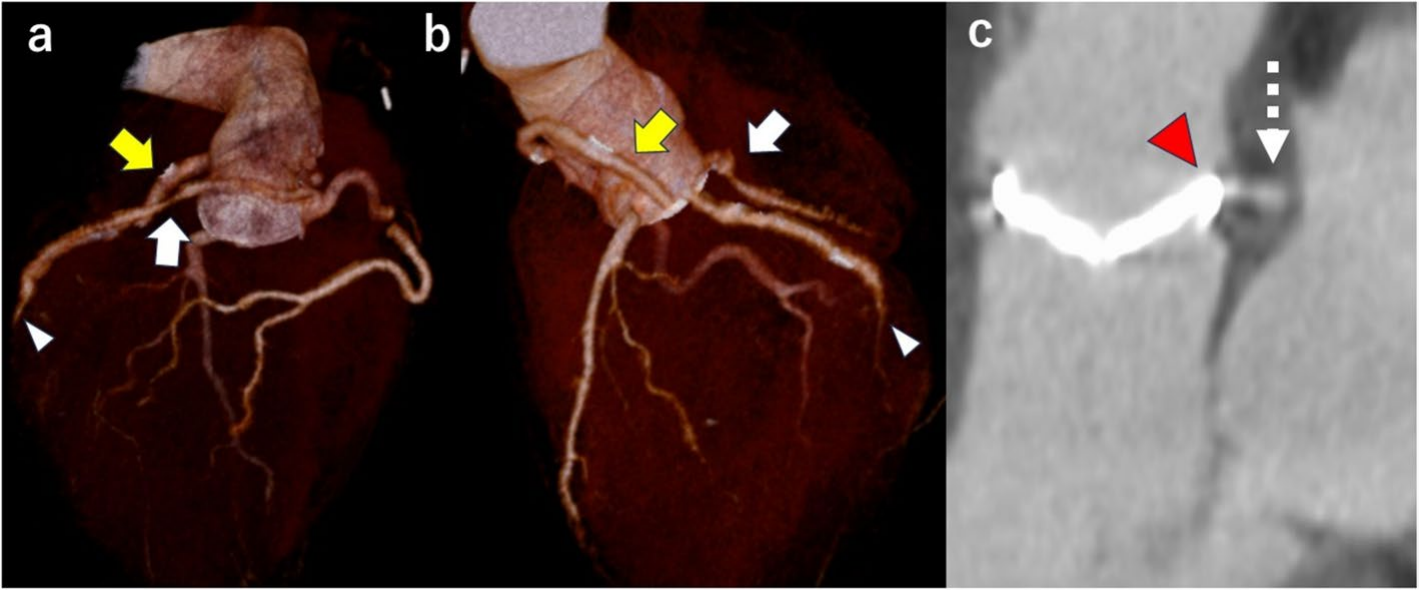
Postoperative computed tomography showing that the bypass graft (yellow arrow) to the obtuse marginal branch (white arrowhead) and anomalous left circumflex coronary artery (white arrow) were patent (**a** and **b**). The prosthetic valve (red arrowhead) and the anomalous left circumflex coronary artery (white dotted arrow) were positioned near each other at the posterior aspect of the aorta (**c**)

The patient’s postoperative course was smooth, and a postoperative CT scan confirmed that the bypass graft and anomalous LCX were patent (Fig. 5a and b). The prosthetic valve and the anomalous LCX were positioned near each other at the posterior aspect of the aorta (Fig. 5c). Transthoracic echocardiography showed trivial MR and no MV aneurysm. The patient was discharged on postoperative day 14 and was living his daily life without any issues while being managed with warfarin and aspirin 6 months postoperatively.

## Discussion and conclusions

Roberts et al. and Veinot et al. reported that AOLCX is a rare cause of intraoperative and remote coronary flow obstruction [1, 2]. Autopsy findings in these cases indicated that the anomalous LCX, running posteriorly along the AV annulus, was compressed by a prosthetic valve, leading to myocardial infarction. Awareness of this pathology has increased, and advancements in imaging techniques have resulted in a growing number of reports on AOLCX [3–19].

Table 1 summarizes previously reported cases of AOLCX, including our own [1–19]. A detailed summary is provided in Additional File 2. The patients ranged from 29 to 87 years of age, with a majority (67%) being male. Most cases involved AV surgery, with a recent increase in reports related to transcatheter AV replacement (TAVR). Notably, AOLCX is more common in patients with BAV.

**Table 1:** Summary of previously reported cases of AOLCX

Variables	Total (*n* = 36)
Age (years)	65 ± 18
Male (*n*, %)	24 (67%)
Affected valve	
Aortic valve (*n*, %)	31 (86%)
Mitral valve (*n*, %)	2 (2.7%)
Aortic and mitral valves (*n*, %)	3 (8.3%)
Bicuspid aortic valve (*n*, %)	16 (44%)
Operative procedure^a^	
Surgical AVR (including Bentall operation) (*n*, %)	19 (53%)
TAVR (*n*, %)	10 (28%)
Double (aortic and mitral) valve replacement (*n*, %)	2 (5.6%)
MVR and MVP (*n*, %)	3 (8.3%)
AVP and remodeling procedure (*n*, %)	3 (8.3%)
Outcomes	
Operative death (*n, %*)	1 (2.8%)
Remote sudden death (*n*)	2
Unexpected LCX blood flow disturbance (*n*, details)	8 (surgical AVR: 4, DVR: 2, MVR: 1, MVP: 1)

*AVP* aortic valve plasty, *AVR* aortic valve replacement, *MVP* mitral valve plasty, *MVR* mitral valve replacement, *LCX* left circumflex coronary artery, *TAVR* transcatheter aortic valve replacement

^a^Duplicates exist

Patients with BAV are typically younger, and preoperative coronary angiography may not be routinely performed. Even when it is performed, the presence of AOLCX may be overlooked [2]. Recent studies have highlighted the utility of multidetector CT scans for the noninvasive detection of AOLCX [5, 6, 11]. For surgical risk assessment, the presence of calcification of the anterior MV annulus and the positioning of the anomalous LCX relative to the AV and MV annulus should be evaluated using CT scans [5, 15].

Regardless of the treatment approach, the careful selection of prosthetic valve size is crucial. Choosing a slightly smaller size is advisable [2, 4–6, 11, 16]. Prosthetic valves with stents and cuffs pose a risk of coronary compression [6]. TAVR valves, whether balloon-expandable or self-expanding, also carry associated risks [9, 10]. The decision between AV and MV repair with a partial ring may be beneficial [6, 10, 12, 14, 17]. A supra-annular position for surgical AV replacement or a higher position for TAVR is recommended [6, 18]. However, adjustments to the fixed position should be made based on the positional relationship of the anomalous LCX to the AV annulus on multidetector CT scans. In this case, intra-annular prosthetic valve implantation with single interrupted sutures could have been an alternative [6].

In surgical AV replacement, if the LCX can be freed, coronary artery entrapment can be avoided through careful needle manipulation [3, 5, 10, 14]. However, dissection of the proximal anomalous LCX may not always be straightforward [11], and preemptive CABG may be warranted in such scenarios [4, 5, 11]. Generally, arterial grafts exhibit a higher patency rate than vein grafts in coronary artery disease but are sensitive to the degree of stenosis in the native coronary artery [20], making their suitability for prophylactic CABG in this context unclear. The use of arterial grafts with ligation of the proximal portion of the anomalous LCX may be considered [5], although no clinical cases have been documented. Conversely, vein grafts are advantageous in situations with a high risk of blood flow competition [20]. Recent reports indicate promising long-term patency rates for no-touch saphenous vein grafts without high-pressure saline distension [21], which may be appropriate in similar cases. In concomitant prophylactic CABG, reducing the size of the prosthetic valve may not be necessary if long-term graft patency can be anticipated [11].

In TAVR, coronary artery stenosis can be identified via coronary angiography, allowing for timely relief of ischemia [9]. However, the absence of coronary artery compression during the procedure does not guarantee a long-term event-free outcome. As with surgical AV replacement, careful follow-up is essential.

In conclusion, we performed preemptive CABG in a patient with AOLCX who underwent AV and MV surgeries. As the incidence of treatments for valvular heart disease rises, more cases of AOLCX may be encountered. It is imperative to accurately diagnose its presence, evaluate treatment risks, select appropriate interventions, and ensure diligent follow-up.

## Supplementary Information

Additional File 1. Intraoperative findings. a) A mitral valve aneurysm (arrowhead) was observed in the clear zone of the anterior leaflet, with no signs of active infection. b) The aneurysm was resected and repaired through direct suturing without the need for mitral annuloplasty.

Additional File 2. A comprehensive summary of previously documented surgical cases involving anomalous origin of the left circumflex coronary artery, alongside our case. ALCX: anomalous left circumflex coronary artery; AR: aortic regurgitation; AS: aortic stenosis; ASR: aortic stenosis and regurgitation; AVP: aortic valve plasty; AVR: aortic valve replacement; BAV: bicuspid aortic valve; CABG: coronary artery bypass grafting; CI: coronary Intervention; D&R: dissection and release; IE: infective endocarditis; MR: mitral regurgitation; MS: mitral stenosis; MV: mitral valve; MVP: mitral valve plasty; MVR: mitral valve replacement; PV: prosthetic valve; TAV: tricuspid aortic valve; TAVR: transcatheter aortic valve replacement; VA-ECMO: venoarterial extracorporeal membrane oxygenation.

## Data Availability

Not applicable.

## Abbreviations

AOLCX: Anomalous origin of the left circumflex coronary artery
AR: Aortic regurgitation
AV: Aortic valve
BAV: Bicuspid aortic valve
CABG: Coronary artery bypass grafting
CT: Computed tomography
LCX: Left circumflex coronary artery
MR: Mitral regurgitation
MV: Mitral valve
RCA: Right coronary artery
TAVR: Transcatheter aortic valve replacement
