# Supplementary figures and images for: Surgical management of a patient with anomalous origin of the left circumflex coronary artery undergoing aortic and mitral valve surgery
Source: Gen Thorac Cardiovasc Surg Cases. 2025 Jul 15;4:32. doi: 10.1186/s44215-025-00215-4 (PMC12261822; doi:10.1186/s44215-025-00215-4)

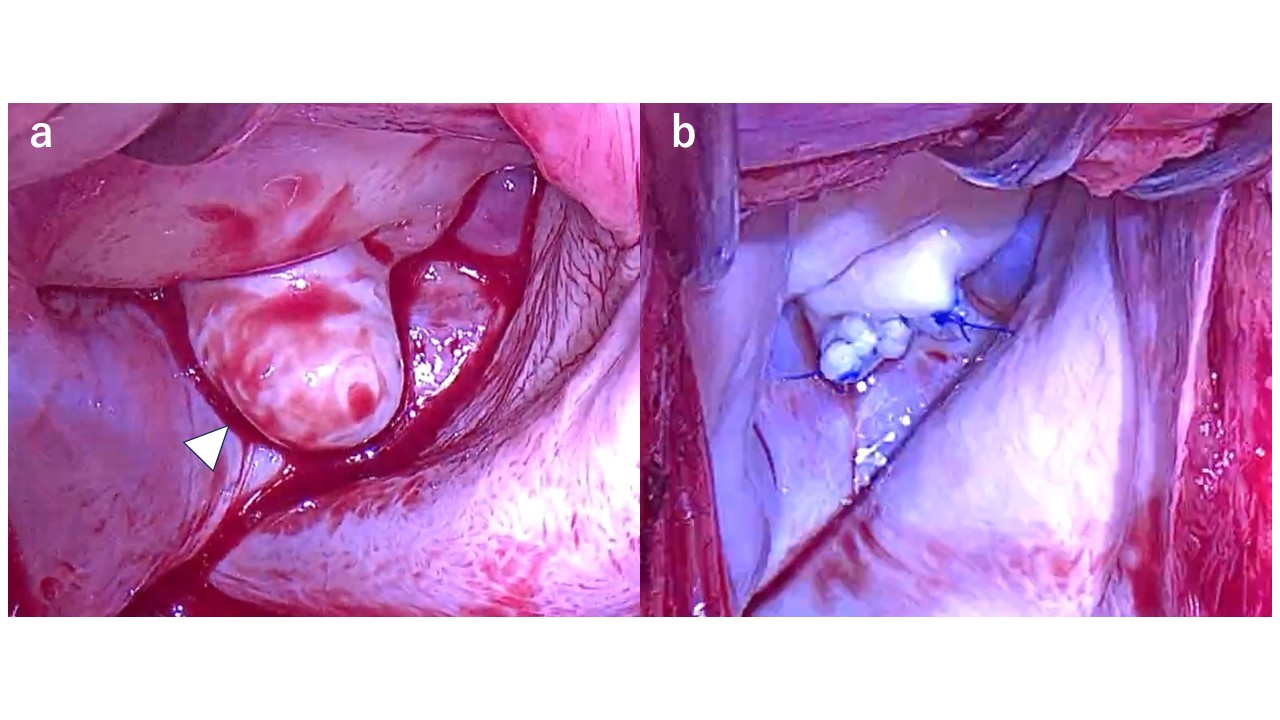

Supplement: Supplementary file 1 — Additional File 1. Intraoperative findings. a) A mitral valve aneurysm (arrowhead) was observed in the clear zone of the anterior leaflet, with no signs of active infection. b) The aneurysm was resected and repaired through direct suturing without the need for mitral annuloplasty. [file 44215_2025_215_MOESM1_ESM.jpg]
