# Supplementary material for: Surgical management of a patient with anomalous origin of the left circumflex coronary artery undergoing aortic and mitral valve surgery
Source: Gen Thorac Cardiovasc Surg Cases. 2025 Jul 15;4:32. doi: 10.1186/s44215-025-00215-4 (PMC12261822; doi:10.1186/s44215-025-00215-4)
Supplement: Supplementary file 2 — Additional File 2. A comprehensive summary of previously documented surgical cases involving anomalous origin of the left circumflex coronary artery, alongside our case. ALCX: anomalous left circumflex coronary artery; AR: aortic regurgitation; AS: aortic stenosis; ASR: aortic stenosis and regurgitation; AVP: aortic valve plasty; AVR: aortic valve replacement; BAV: bicuspid aortic valve; CABG: coronary artery bypass grafting; CI: coronary Intervention; D&R: dissection and release; IE: infective endocarditis; MR: mitral regurgitation; MS: mitral stenosis; MV: mitral valve; MVP: mitral valve plasty; MVR: mitral valve replacement; PV: prosthetic valve; TAV: tricuspid aortic valve; TAVR: transcatheter aortic valve replacement; VA-ECMO: venoarterial extracorporeal membrane oxygenation. [file 44215_2025_215_MOESM2_ESM.pptx]

## Slide 1
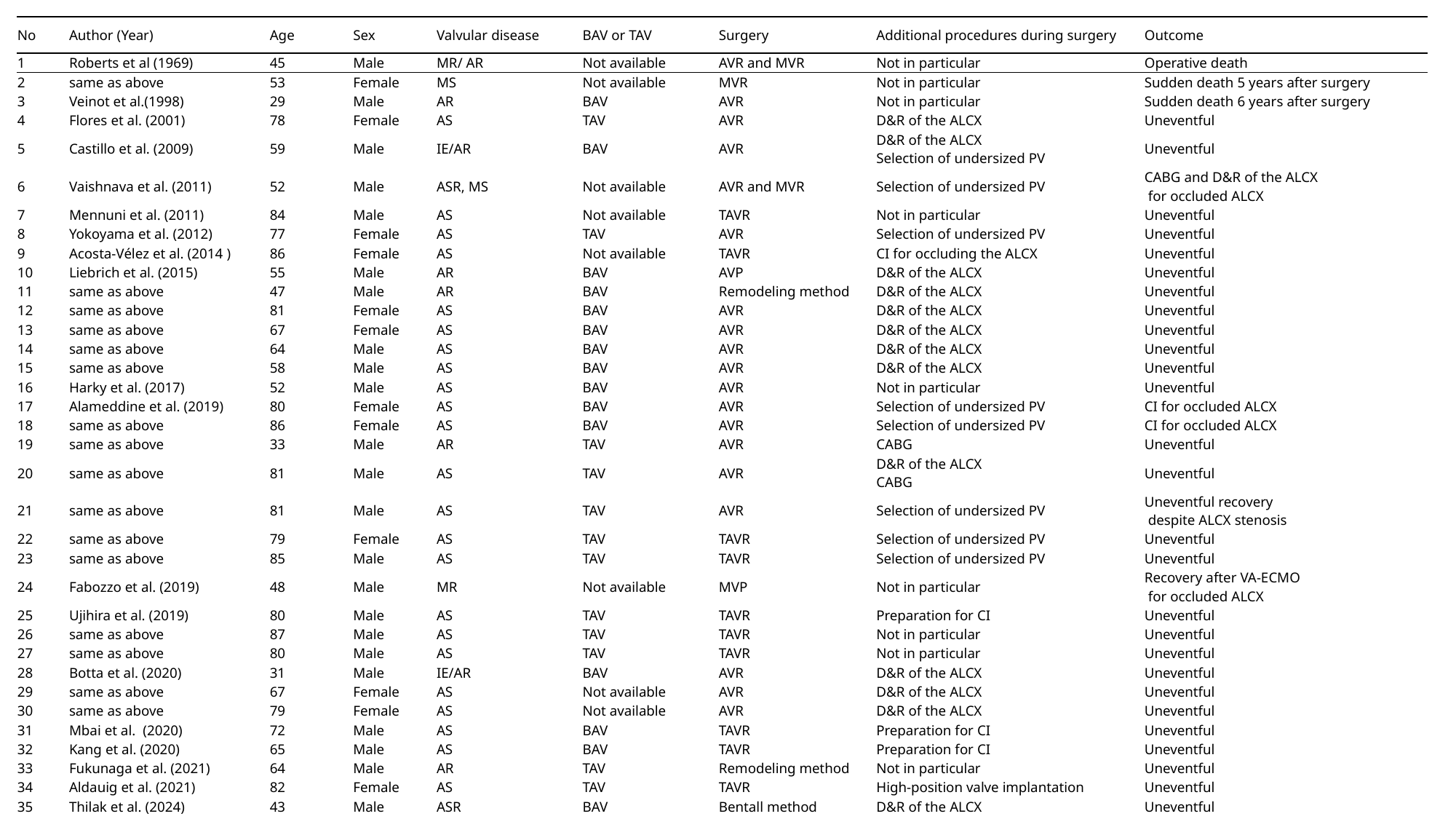

| No | Author (Year) | Age | Sex | Valvular disease | BAV or TAV | Surgery | Additional procedures during surgery | Outcome |
| --- | --- | --- | --- | --- | --- | --- | --- | --- |
| 1 | Roberts et al (1969) | 45 | Male | MR/ AR | Not available | AVR and MVR | Not in particular | Operative death |
| 2 | same as above | 53 | Female | MS | Not available | MVR | Not in particular | Sudden death 5 years after surgery |
| 3 | Veinot et al.(1998) | 29 | Male | AR | BAV | AVR | Not in particular | Sudden death 6 years after surgery |
| 4 | Flores et al. (2001) | 78 | Female | AS | TAV | AVR | D&R of the ALCX | Uneventful |
| 5 | Castillo et al. (2009) | 59 | Male | IE/AR | BAV | AVR | D&R of the ALCX Selection of undersized PV | Uneventful |
| 6 | Vaishnava et al. (2011) | 52 | Male | ASR, MS | Not available | AVR and MVR | Selection of undersized PV | CABG and D&R of the ALCX for occluded ALCX |
| 7 | Mennuni et al. (2011) | 84 | Male | AS | Not available | TAVR | Not in particular | Uneventful |
| 8 | Yokoyama et al. (2012) | 77 | Female | AS | TAV | AVR | Selection of undersized PV | Uneventful |
| 9 | Acosta-Vélez et al. (2014 ) | 86 | Female | AS | Not available | TAVR | CI for occluding the ALCX | Uneventful |
| 10 | Liebrich et al. (2015) | 55 | Male | AR | BAV | AVP | D&R of the ALCX | Uneventful |
| 11 | same as above | 47 | Male | AR | BAV | Remodeling method | D&R of the ALCX | Uneventful |
| 12 | same as above | 81 | Female | AS | BAV | AVR | D&R of the ALCX | Uneventful |
| 13 | same as above | 67 | Female | AS | BAV | AVR | D&R of the ALCX | Uneventful |
| 14 | same as above | 64 | Male | AS | BAV | AVR | D&R of the ALCX | Uneventful |
| 15 | same as above | 58 | Male | AS | BAV | AVR | D&R of the ALCX | Uneventful |
| 16 | Harky et al. (2017) | 52 | Male | AS | BAV | AVR | Not in particular | Uneventful |
| 17 | Alameddine et al. (2019) | 80 | Female | AS | BAV | AVR | Selection of undersized PV | CI for occluded ALCX |
| 18 | same as above | 86 | Female | AS | BAV | AVR | Selection of undersized PV | CI for occluded ALCX |
| 19 | same as above | 33 | Male | AR | TAV | AVR | CABG | Uneventful |
| 20 | same as above | 81 | Male | AS | TAV | AVR | D&R of the ALCX CABG | Uneventful |
| 21 | same as above | 81 | Male | AS | TAV | AVR | Selection of undersized PV | Uneventful recovery despite ALCX stenosis |
| 22 | same as above | 79 | Female | AS | TAV | TAVR | Selection of undersized PV | Uneventful |
| 23 | same as above | 85 | Male | AS | TAV | TAVR | Selection of undersized PV | Uneventful |
| 24 | Fabozzo et al. (2019) | 48 | Male | MR | Not available | MVP | Not in particular | Recovery after VA-ECMO for occluded ALCX |
| 25 | Ujihira et al. (2019) | 80 | Male | AS | TAV | TAVR | Preparation for CI | Uneventful |
| 26 | same as above | 87 | Male | AS | TAV | TAVR | Not in particular | Uneventful |
| 27 | same as above | 80 | Male | AS | TAV | TAVR | Not in particular | Uneventful |
| 28 | Botta et al. (2020) | 31 | Male | IE/AR | BAV | AVR | D&R of the ALCX | Uneventful |
| 29 | same as above | 67 | Female | AS | Not available | AVR | D&R of the ALCX | Uneventful |
| 30 | same as above | 79 | Female | AS | Not available | AVR | D&R of the ALCX | Uneventful |
| 31 | Mbai et al. (2020) | 72 | Male | AS | BAV | TAVR | Preparation for CI | Uneventful |
| 32 | Kang et al. (2020) | 65 | Male | AS | BAV | TAVR | Preparation for CI | Uneventful |
| 33 | Fukunaga et al. (2021) | 64 | Male | AR | TAV | Remodeling method | Not in particular | Uneventful |
| 34 | Aldauig et al. (2021) | 82 | Female | AS | TAV | TAVR | High-position valve implantation | Uneventful |
| 35 | Thilak et al. (2024) | 43 | Male | ASR | BAV | Bentall method | D&R of the ALCX | Uneventful |
| 36 | our case | 36 | Male | AR, MV anuerysm | BAV | AVR and MVP | Selection of undersized PV CABG | Uneventful |
